# Investigation and Analysis of Sharp Injuries among Health Care Workers from 36 Hospitals in Shandong Province, China

**DOI:** 10.1155/2021/5698483

**Published:** 2021-06-11

**Authors:** Jian Sun, Wen Qin, Lei Jia, Zhen Sun, Hua Xu, Yiyi Hui, Anman Gu, Weiguang Li

**Affiliations:** ^1^Department of Infection Control, Shandong Provincial Hospital Affiliated to Shandong First Medical University, Jinan, Shandong 250021, China; ^2^Department of Infection Control, The Affiliated Hospital of Qingdao University, Qingdao 266062, China

## Abstract

**Background:**

This study investigated and analyzed the current situation of sharp injuries among health care workers (HCWs) in China's Shandong Province.

**Methods:**

By means of questionnaire survey, the incidence of sharp injuries among HCWs from 36 hospitals in China's Shandong Province in October 2019 was investigated, and the results of this survey were compared with those of October 2012.

**Results:**

A total of 48165 HCWs were investigated. 549 cases of sharp injuries occurred. The incidence of sharp injuries was 1.14%, which was significantly lower than that in October 2012 (9.71%). In the occupational distribution of sharp injuries among HCWs, the proportion of nurses was 58.65%, doctors 23.32%, and interns 12.02%. Among the distribution of sharp injury departments, general wards, operating rooms, intensive care units, disinfection supply centers, and outpatient clinics were the high-incidence sites of occupational exposure among HCWs. The main instruments causing sharp injuries in HCWs were syringes, scalp steel needles, surgical suture needles, vacuum blood collection needles, and glass slides. Drug administration, double-handed loop needle cap, blood extraction, surgical suture needle, and arteriovenous needle extraction were high-risk operations causing sharp instrument injuries in HCWs.

**Conclusion:**

The incidence of sharp injuries among HCWs from 36 hospitals in Shandong Province in October 2019 was significantly lower than that in October 2012. Sharp injuries were a common type of occupational exposure for HCWs. The occurrence of sharp injuries should be effectively reduced by changing wrong habitual behavior and implementing standard protective measures.

## 1. Introduction

Professional exposure to sharp injuries among HCWs refers to the occupational injuries caused by various sharp instruments such as needles, knives, and glass fragments piercing the skin during the process of medical treatment [1].

About 3.35 million HCWs suffer from sharp injuries every year around the world [2]. It has been estimated that a HCW may suffer from sharp injuries 4 times every year in Africa, the Eastern Mediterranean, and Asia [3]. The Centers for Disease Control and Prevention estimate that 385,000 sharp injuries occur among HCWs in U.S. hospitals per year [4]. The World Health Organization (WHO) estimates suggest that 1 in 10 HCWs worldwide sustains a sharp injury each year [5]. In Egypt, a study conducted in Gharbiya Governorate showed that 66.2% of HCWs reported that they experienced at least one sharp injury in their working life [6]. Another study which was conducted at the 3 teaching hospitals of Alexandria University reported that 67.9% of HCWs had at least 1 sharp injury in the previous 12 months [7].

The report of sharp injury is the main way for the competent department to find sharp injury events. The probability of corresponding infection is small after being stabbed by a contaminated sharp instrument [2]. Once infection occurs, the consequences are serious. Timely reporting and corresponding interventions can effectively reduce the risk of infection after exposure.

Although sharp injuries are one of the most common types of injury reported by HCWs, it is believed that they are vastly underreported [8]. Various studies indicate that underreporting of sharp injuries is prevalent in health care facilities worldwide, with rates of underreporting ranging from 19% to 86% [9, 10]. Prompt reporting of needlestick injuries is important, not only for the management of the exposure (the efficacy of PEP regimens is approximately 81% for HIV8 and 85%-95% for HBV 9) but also for identification of workplace hazards and evaluation of prevention measures [2].

Only a few studies have been published on sharp injuries in developing countries [11–13]. In China, HCWs are facing a high risk of sharp injuries, and the situation is grim. Although HCWs' understanding of occupational exposure has changed greatly after SARS and hospitals have also used various forms of occupational protection training and formulated corresponding systems and protective measures, still, it is difficult to resist the harm of occupational exposure to HCWs. The objectives of this study were to determine the incidence of occupational exposure and protection against sharp injuries among HCWs in Shandong Province during October 2019 and also to compare the current data with the October 2012 data.

## 2. Material and Methods

### 2.1. Investigation Design

An investigation about the occurrence of sharp injuries among HCWs was conducted in 36 public hospitals in China's Shandong Province in October 2019. Prior to this, the same investigation was carried out in these hospitals in October 2012.

### 2.2. Investigation Methods

By means of questionnaire survey, the occurrence of sharp injuries among HCWs in 36 hospitals in Shandong Province in October 2019 was investigated. The questionnaire was a slightly modified form based on the “2010 Needlestick and Sharp Object Injury Report Form” of EPINet®, which consisted of 20 variables grouped in blocks (general and individual information, the source of exposure, occurrence time, occurrence place, involved operation, occurrence department, and whether to report or not). The results of this survey were compared with those of October 2012. The two surveys were conducted in the same 36 hospitals, and all HCWs in each hospital participated in the survey. Due to the reasons of new entry, retirement, and resignation, the HCWs of each hospital changed, but the change rate was less than 3%. And the scale of each hospital did not change significantly.

### 2.3. Sampling Method and Study Population

A clustered random sampling method was conducted to perform the questionnaire among HCWs from 36 hospitals in Shandong Province. The sample size was determined from a prevalence estimate of 10% and a population of 370,000 to give a confidence level of 0.95 and precision of 2.5 to be 554 (Epitools epidemiological calculators, Ausvet Pty Ltd., available at https://epitools.ausvet.com.au). In order to reduce the sampling error and increase the accuracy of the survey and the representativeness of the sample, we increased the sample size. 50618 questionnaires were sent out, and 48165 valid questionnaires were collected.

### 2.4. Informed Consent and Ethical Review

The survey was officially approved by the participating hospitals, and an agreement was signed with each hospital. The participating hospitals informed the investigated HCWs of the objectives, contents, and procedures of the survey and obtained the HCWs' consent. The survey was anonymous and did not involve the identity information of participants. This study was designed as a retrospective study, and collected data were confidentially kept. Therefore, it was exempt from ethical review.

### 2.5. Data Quality Control

These two investigations were both carried out with the same survey method in China's Shandong Province. Uncertain or incomplete data was eliminated to ensure the accuracy and integrity of data. The incidence of sharp injuries in this study refers to the proportion of people who suffered from sharp injuries in the last month (October 2019 or October 2012).

### 2.6. Statistical Analysis

Data analyses were made using SPSS Statistics V20.0. The survey results were input into SPSS 20.0 software, and the Pearson *X*^2^ test was used to analyze the relevant data. The test level was set as alpha = 0.05.

## 3. Results

### 3.1. Incidence of Sharp Injuries

A total of 48165 HCWs were surveyed. 549 cases of sharp injuries occurred. The incidence of sharp injuries was 1.14%, which was significantly lower than that in 2012 (9.71%). The specific data is shown in Table 1.

### 3.2. Profession Distribution of Sharp Injuries

Nurses (322 cases, 58.65%) were the major components of sharp injuries, followed by doctors (128 cases, 23.32%) and interns (66 cases, 12.02%). Incidences of sharp injuries in various occupations are shown in Table 2.

### 3.3. Locations of Sharp Injury Events

Professional exposure to sharp injuries may occur in all kinds of hospital environments. This survey found that general wards, operating rooms, intensive care units, disinfection supply centers, and outpatient clinics were the top five locations in which HCWs were exposed to sharp injuries. The specific data is shown in Table 3.

### 3.4. Tools That Caused Injuries

In the survey, we found that the top five tools causing sharp injuries were syringes, scalp steel needles, surgical suture needles, vacuum blood collection needles, and glass slides, as shown in Table 4.

### 3.5. Operations That Caused Sharp Injuries

In the survey, it was found that the top five operations causing sharp injuries were recapping injector needle with two hands, drug administration, drawing blood, surgical suture, and withdrawing needles, as shown in Table 5.

### 3.6. Report of Sharp Injuries

In the survey, the highest report rate of sharp injuries was technicians 64.71%, followed by doctors 38.28%, nurses 34.16%, and interns 31.82%. The overall report rate of sharp injuries among HCWs was 35.34% in Shandong Province in October 2019, which was significantly higher than that in 2012 (15.75%). The specific data is shown in Table 6.

## 4. Discussion

The results of this survey showed that the incidence of sharp injuries among HCWs in Shandong Province was 1.14% in October 2019, which was basically consistent with the reports in other regions of China [14–16], lower than those in other countries [17–21]. On the one hand, government and medical institutions provided a lot of educational activities in recent years, the awareness of occupational exposure and protection among HCWs has increased significantly, and the protective equipment has been improved, so the incidence of sharp injuries has decreased; on the other hand, the HCWs involved in the survey failed to report or conceal sharp injury events for various reasons, which also led to a low incidence.

The incidence of sharp injuries in this survey was significantly lower than that in 2012, and incidences of sharp injuries in various occupations in October 2019 were also significantly lower than those in 2012. The survey results sufficiently demonstrated that the current situation of occupational exposure and protection of HCWs in Shandong Province had gradually improved.

Occupational exposure of HCWs may occur in any environment of the hospital. This current study found that general ward, operating room, and ICU were the top three departments of occupational exposure of HCWs, which was consistent with the reports in other regions of China [14, 15]. The operations prone to sharp injuries were mainly recapping injector needle with two hands, drug administration, and drawing blood, which was consistent with the reports in China [22]. Tools that caused sharp injuries among HCWs were mainly syringes, scalp steel needles, and surgical suture needles, which were basically consistent with the relevant reports at home and abroad [16, 17, 22].

However, there are still some problems in occupational exposure and protection of HCWs in Shandong Province. Firstly, the use rate of safety devices is low. Since the first safety devices came out in 1988, many studies have proved that safety devices can reduce sharp injuries of HCWs [23–26]. Although there are many types of occupational exposure among HCWs, sharp injuries are the most common and harmful [27]. A survey from China Hospital Association shows that although safety devices had been gradually promoted and used throughout the country in recent years, only 52.53% of hospitals were trying to use safety devices, and the utilization rate of safety devices is still low [22].

Secondly, the behaviors of HCWs were not standardized. Relevant studies showed that most nurses exposed to sharp injuries did not follow the relevant regulations and suggestions to prevent needlestick injuries during operation, and the incidence of sharp injuries was higher while disposing sharp instruments [28]. Gershon et al. showed that the incidence of sharp injuries was related to recapping injector needle [29]. In this survey, it was also found that recapping injector needle was a high-risk operation for sharp injuries, which was consistent with the above reports.

Thirdly, the report rate of sharp injuries is low. After occupational exposure occurs, the exposed person should report immediately and get further emergency treatment. Hospitals should establish appropriate emergency response mechanisms so that exposures can be timely reported and treated and receive regular monitoring and follow-up [30, 31]. In Salelkar et al.'s report, the report rate of sharp injuries was 32% [32], in Khader et al.'s report 22.9% [33], and in this survey 35.34%, which is basically the same as the above reports. The most common reason for failing to report is the lack of awareness among HCWs [22].

In the case of sharp instrument injury contaminated by blood-borne pathogens, the corresponding infection rate is estimated to be as follows: hepatitis B 3–10%; hepatitis C 0.8–3%; and HIV 0.3% [34]. Hospitals should establish corresponding emergency response mechanisms so that exposures can be reported and dealt with in a timely manner. In view of the risk of postsharp injury infection, medical and health institutions should assess the risk of infection based on the available information, including the results of examinations of the source patients and types and duration of occupational exposure; assess and determine the level of exposure and the level of infectious pathogenicity of the source of exposure; and determine whether to use preventive drugs and what preventive measures to take. The case should have been followed up. In view of the psychological barriers of HCWs after sharp injuries [35], we should also pay attention to the changes of psychological status of HCWs and provide timely psychological support and intervention to ensure the physical and mental health of HCWs.

## 5. Conclusion and Limitations

There were some limitations to this study. First, it relied on the respondents' questionnaire data, which were subject to subjective consciousness and recall bias. This could have resulted in an underestimation of actual incidence of sharp injuries. In addition, the incidence of sharp injuries could vary among hospitals due to the difference in safety measures, reporting systems, and facility appliances. Due to the limitation of the survey content, it was difficult to compare and analyze the differences among these hospitals.

The incidence of sharp injuries among HCWs from 36 hospitals in Shandong Province in October 2019 was significantly lower than that in October 2012. Sharp injuries were a common type of occupational exposure for HCWs. The occurrence of sharp injuries should be effectively reduced by changing wrong habitual behavior and implementing standard protective measures.

## Figures and Tables

**Table 1:** The incidence of sharp injuries among HCWs in Shandong Province.

Survey time	Survey number	Number of sharp injuries	Incidence^a^ (%)	*P*
October 2019	48165	549	1.14	<0.05
October 2012	46600	4526	9.71	

^a^Incidence of sharp injuries refers to the proportion of people who suffered from sharp injuries in the last month (October 2019 or October 2012). *P* < 0.05: statistically significant.

**Table 2:** Incidences of sharp injuries in various occupations in Shandong Province in October 2012 and October 2019.

Profession	Survey number		Number of sharp injuries		Incidence (%)		*P*
	October 2019	October 2012	October 2019	October 2012	October 2019	October 2012	
Nurse	21953	21825	322	2918	1.47	13.37	<0.05
Doctor	18420	13746	128	916	0.69	6.66	<0.05
Intern	2320	4048	66	496	2.84	12.25	<0.05
Technician	2043	1993	17	58	0.83	2.91	<0.05
Cleaning staff	2578	2917	12	98	0.47	3.36	<0.05
Other	851	2071	4	40	0.47	1.93	<0.05
Total	48165	46600	549	4526	1.14	9.71	<0.05

*P* < 0.05: statistically significant.

**Table 3:** Locations of sharp injuries among HCWs in Shandong Province in October 2019.

Department	Number of sharp injuries		Proportion (%)		*P*
	October 2019	October 2012	October 2019	October 2012	
General wards	245	2242	44.63	49.54	<0.05
Operating rooms	98	855	17.85	18.89	>0.05
ICU^a^	37	236	6.74	5.21	>0.05
CSSD^b^	26	80	4.74	1.77	<0.05
Outpatient dept	23	116	4.19	2.56	<0.05
Emergency dept	21	121	3.83	2.67	>0.05
Blood-drawing dept	15	31	2.73	0.68	<0.05
Medical waste repository	14	43	2.55	0.95	<0.05
Rehydration room	10	101	1.82	2.23	>0.05
Clinical laboratory	5	41	0.91	0.91	>0.05
PIVAS^c^	4	38	0.73	0.84	>0.05
Dental dept	4	146	0.73	3.23	<0.05
Hemodialysis room	3	13	0.55	0.29	>0.05
Ophthalmology dept	3	34	0.55	0.75	>0.05
Other	41	429	7.47	9.48	>0.05
Total	549	4526	100.00	100.00	

^a^ICU: intensive care units; ^b^CSSD: central sterile supply department; ^c^PIVAS: pharmacy intravenous admixture service. *P* < 0.05: statistically significant.

**Table 4:** Tools that caused sharp injuries among HCWs in Shandong Province in October 2019.

Tool	Number of sharp injuries		Proportion (%)		*P*
	October 2019	October 2012	October 2019	October 2012	
Syringes	199	1578	36.25	34.87	>0.05
Scalp steel needles	110	1049	20.04	23.18	>0.05
Surgical suture needles	68	403	12.39	8.90	<0.05
Blood collection needles	26	90	4.74	1.99	<0.05
Glass slides	20	55	3.64	1.22	<0.05
Vein detained needle	17	141	3.10	3.12	>0.05
Scalpel	13	176	2.37	3.89	>0.05
Sectioning knife	6	44	1.09	0.97	<0.05
Scissors	5	40	0.91	0.88	>0.05
Puncture needle	3	23	0.55	0.51	>0.05
Other	82	927	14.94	20.49	<0.05
Total	549	4526	100.00	100.00	

*P* < 0.05: statistically significant.

**Table 5:** Operations that caused sharp injuries among HCWs in Shandong Province in October 2019.

Operation	Number of sharp injuries		Proportion (%)		*P*
	October 2019	October 2012	October 2019	October 2012	
Recapping injector needle with two hands	90	467	16.39	10.32	<0.05
Drug administration	82	508	14.94	11.22	<0.05
Drawing blood	51	173	9.29	3.82	<0.05
Surgical suture	49	397	8.93	8.77	>0.05
Withdrawing needles	36	371	6.56	8.20	>0.05
Tidying up surgical instruments	35	155	6.38	3.42	<0.05
Putting needles into the sharp box	29	317	5.28	7.00	>0.05
Preparation for infusion	27	278	4.92	6.14	>0.05
Intravenous injection	25	229	4.55	5.06	>0.05
Handling trash	24	346	4.37	7.64	<0.05
Needle or instrument delivery	19	113	3.46	2.50	>0.05
Contusion during rescue	14	124	2.55	2.74	>0.05
Intramuscular injection	13	113	2.37	2.50	>0.05
Draw arterial blood	9	72	1.64	1.59	>0.05
Surgical knife cuts	6	122	1.09	2.70	<0.05
Other	40	741	7.29	16.37	<0.05
Total	549	4526	100.00	100.00	

*P* <0.05: statistically significant.

**Table 6:** Report rates of sharp injuries among HCWs in Shandong Province in October 2019 and October 2012.

Profession	Sharp injuries		Reported number		Report rate (%)		*P*
	October 2019	October 2012	October 2019	October 2012	October 2019	October 2012	
Technician	17	58	11	12	64.71	20.69	<0.05
Doctor	128	916	49	152	38.28	16.59	<0.05
Nurse	322	2918	110	430	34.16	14.73	<0.05
Intern	66	496	21	88	31.82	17.76	<0.05
Other	5	40	1	9	20.00	22.50	>0.05
Cleaning staff	11	98	2	22	18.18	22.45	>0.05
Total	549	4526	194	713	35.34	15.75	<0.05

*P* < 0.05: statistically significant.

## Data Availability

The data used to support the findings of this study are included within the article.
